# Embryotoxic and Teratogenic Effects of Norfloxacin in Pregnant Female Albino Rats

**DOI:** 10.1155/2014/924706

**Published:** 2014-02-03

**Authors:** Mohamed Aboubakr, Mohamed Elbadawy, Ahmed Soliman, Mohamed El-Hewaity

**Affiliations:** ^1^Department of Pharmacology, Faculty of Veterinary Medicine, Benha University, Moshtohor, Toukh, Qaliobiya 13736, Egypt; ^2^Department of Pharmacology, Faculty of Veterinary Medicine, Cairo University, Giza 12211, Egypt; ^3^Department of Pharmacology, Faculty of Veterinary Medicine, University of Sadat City, Minoufiya 32897, Egypt

## Abstract

This study was designed to investigate the possible developmental teratogenicity of norfloxacin in rats. Forty pregnant female rats were divided into four equal groups. Group A received norfloxacin in a dose of 500 mg/kg·b·wt/day orally from 6th to 15th day of gestation. Groups B and C received 1000 and 2000 mg/kg·b·wt/day orally for the same period, respectively; Group D behaved as control and received 0.5 mL distilled water orally for the same period. The dams were killed on 20th day of gestation and their fetuses were subjected to morphological, visceral, and skeletal examinations. Norfloxacin significantly decreased the number of viable fetuses, increased the number of resorbed fetuses, and induced retardation in growth of viable fetuses; some visceral and skeletal defects in these fetuses were seen and these effects were dose dependant. Conclusively, norfloxacin caused some fetal defects and abnormalities, so it is advisable to avoid using this drug during pregnancy.

## 1. Introduction

Teratology, the study of abnormal prenatal development and congenital malformations induced by exogenous chemical or physical agents, is a growing area of medical research in the quest for the eradication of preventable birth defects. Birth defects are known to occur in huge numbers; roughly 7~10% of all children require extensive medical care to diagnose or treat a birth defect; this compromises the quality of life of millions of people worldwide [1]. Almost all therapeutic agents cross placental barrier and enter fetal circulation. Every agent given during pregnancy therefore has a tendency to produce some sort of structural abnormality in the neonate at birth until proved otherwise [2]. A birth defect or a congenital malformation is a structural abnormality of any type present at birth. It may be macroscopic or microscopic, on the surface or within the body [3]. During the past few decades, it has become increasingly evident that human and animal embryos are subjected to the toxic effects of many drugs, such as the use of some antibiotics in the treatment of serious diseases occurring during pregnancy. Fluoroquinolones are one of the main classes of antimicrobials used in treatment of many infections including urinary, respiratory, gastrointestinal tract, skin, bone, and joint infections [4, 5].

The popularity of fluoroquinolone antibiotics has increased because of their broad antimicrobial spectrum, multiple approved indications, and favorable pharmacokinetics [6]. Norfloxacin is synthetic antimicrobial agent of the fluoroquinolones class. Like other fluoroquinolones, norfloxacin acts principally by inhibition of DNA gyrase, an enzyme required for the proper supercoiling of bacterial chromosomes [7]. Norfloxacin is active mainly against Gram-negative and Gram-positive pathogens. It has a wide spectrum of activity and is rapidly bactericidal at low concentration [8].

Norfloxacin is mainly used for the treatment of urinary tract infections which have high incidence during pregnancy especially in the first trimester. With this objective in view, the present study was done to demonstrate the teratogenic effects of norfloxacin in albino rats.

## 2. Materials and Methods

### 2.1. Drug

Norfloxacin was obtained as an oral solution from ATCO Pharma for pharmaceutical industries, Egypt, under a trade name (Atonor). Each mL contains 300 mg of norfloxacin base.

### 2.2. Experimental Animals

Forty mature healthy female albino rats were obtained from department of Laboratory Animal Colonies, Ministry of Public Health, Helwan, Egypt. Animals were kept under hygenic conditions and fed on balanced ration and water *ad libitum*. Female rats were examined periodically using vaginal smear test to ensure that they were always in regular oestrous cycle [9]. They were kept with normal healthy male albino rats allowing one male for two female rats in one cage overnight [10]. The presence of sperms in the vagina next morning was considered as the first day of pregnancy [11]. Pregnancy was confirmed by persistence of diestrus state for 5 days after mating.

### 2.3. Experimental Design

The pregnant rats were divided into four groups each of 10 rats. Rats were given norfloxacin orally from 6th to 15th day of gestation.

- Group A, received norfloxacin orally at a dose of 500 mg/kg·b·wt/day.
- Group B, received norfloxacin orally at a dose of 1000 mg/kg·b·wt/day.
- Group C, received norfloxacin orally at a dose of 2000 mg/kg·b·wt/day.
- Group D, behaved as control group and received 0.5 mL of distilled water orally for the same period.

The drug was given from 6th to 15th day of gestation during the period of fetal organogenesis. All females were killed on the 20th day of pregnancy and their uteri were dissected in order to examine the position and number of viable, resorbed, or dead fetuses. The surviving fetuses were weighed and the length from crown to rump was measured and examined for any external gross malformations, while others were stained by alizarin red for skeletal examination [12]. Cross-sections through the spinal cord and thoracic vertebrae of fetus at 20th day of gestation were done and stained with haematoxylin and eosin for histopathological examinations [13].

## 3. Results

**Figure 1:**
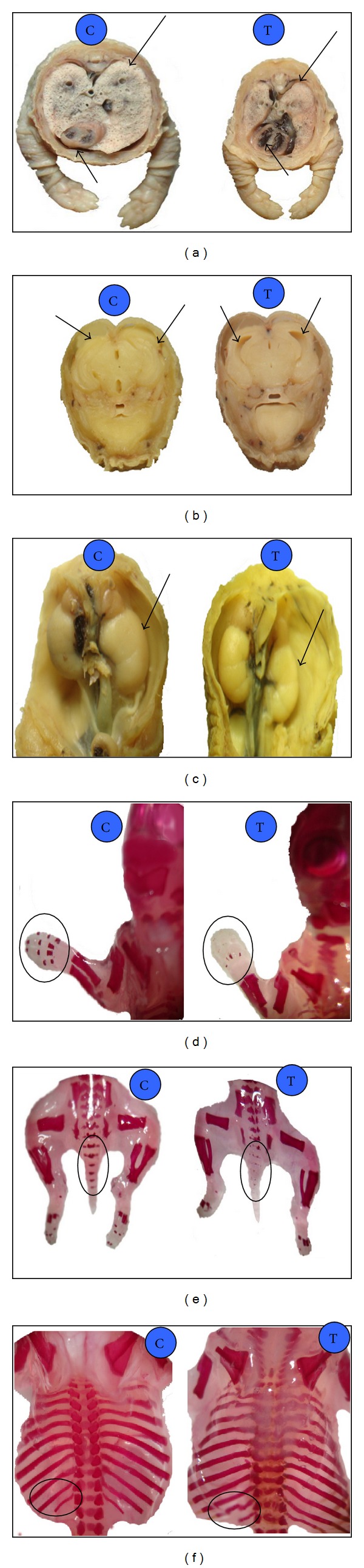
(a) Pulmonary hypoplasia with cardiac enlargement, (b) diverticulum dilatation, (c) kidney hypoplasia, (d) absence of digit's bone of fore limb, (e) partial absence of caudal vertebrae, and (f) irregular and short ribs of a fetuses obtained from pregnant rats after repeated oral administration of 2000 mg norfloxacin/Kg·b·wt from 6th to 15th day of pregnancy. C in blue circle represent (control group) and T in blue circle represent (treated group).

Oral administration of norfloxacin in different doses (500, 1000 and 2000 mg/Kg·b·wt) to pregnant female rats from 6th to 15th days of pregnancy induced changes in number of viable, dead, and resorbed fetuses, fetal body weight, and crown-rump length which were recorded in Table 1. Visceral abnormalities of fetuses were recorded (Table 2 and Figures 1(a), 1(b), and 1(c)), while skeletal examination of alizarin red stained fetuses showed different abnormalities (Table 3 and Figures 1(d), 1(e), and 1(f)).

**Figure 2:**
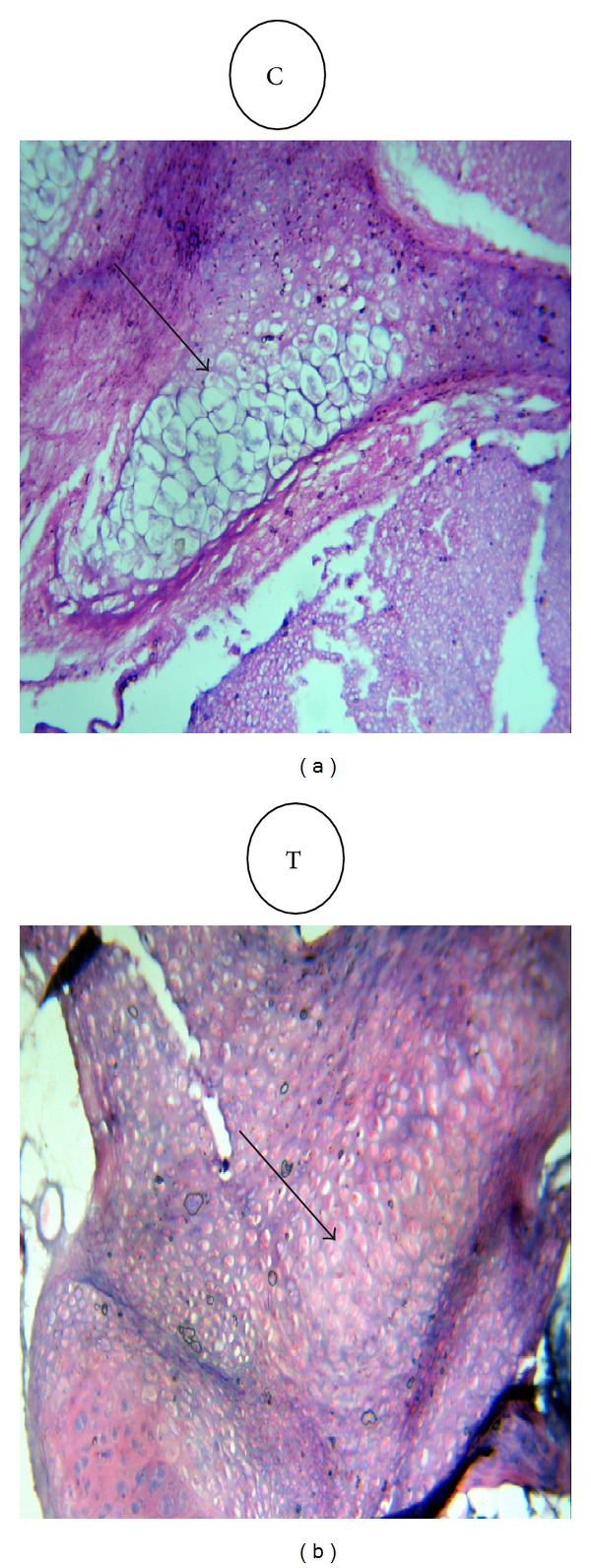
Cross-section through the spinal cord and thoracic vertebrae of fetuses at 20th day of gestation, showing absence of ossification in treated group (T) (2000 mg norfloxacin/Kg·b·wt from 6th to 15th day of pregnancy) in comparison with normal ossification in control group (C) (×10 H & E).

Histopathological examination of fetuses bone (spinal cord and thoracic vertebrae) at 20th day of gestation showed absence of ossification especially in treated group (2000 mg/kg·b·wt) in comparison with normal ossification in control group which was shown in Figure 2.

## 4. Discussion

Oral administration of norfloxacin to female pregnant rats induced decrease in the number of fetuses and increase in the number of resorbed fetuses either early or late when compared with that recorded value of the control group. This result was consistent with the data reported after administration of enrofloxacin, ciprofloxacin, ofloxacin, and norfloxacin to domestic animals, where embryonic losses in female monkeys exposed to very high doses led to decrease in number of the fetuses [14]. The decrease in number of fetuses per mother might be attributed to the lack of oval production or lack of the basic cell constituent as a result of drug administration [15]. Decrease in number of viable fetuses might be explained on the basis of incomplete formation of the placenta and degeneration of the trophoblast and decidual cell, which play an important role in the transmission of nutrients to the embryo [16]. Also, the reduction in number may be due to early embryonic death and an increase in the fetal resorption ratio in the present study.

Administration of norfloxacin to female pregnant rats during the period of organogenesis produced significant decrease in both weight and length of fetuses. These results were consistent with those previously reported following administration of ciprofloxacin to albino rats [17]. These changes may be attributed to deficiency of nutritional supply from dam to fetuses because female rats receiving ofloxacin or levofloxacin exhibited soft stool or diarrhea which might be attributed to imbalance in intestinal microflora [18, 19].

Norfloxacin resulted in many visceral abnormalities as diverticulum dilatation in the brain of fetuses which might be attributed to the lack of placental transfusion of amino acid, arginine, metabolism in fetus [15], neurotoxic effect of norfloxacin [20], or some antibacterials that had neurotoxic effect as levofloxacin and ciprofloxacin which easily cross blood brain barrier and compete with gamma-aminobutyric acid receptor [21]. Norfloxacin induced a hypoplasia or absence of thymus gland of fetuses; this fetal abnormality agreed with the results reported after administration of ciprofloxacin and ofloxacin at a dose of 100 mg/mL, which inhibited the cell growth, while 1000 mg/mL led to cell death [22]. Activity of ciprofloxacin against calf thymus and cultured mammalian cell was studied and this result might be attributed to cytotoxicity of quinolone as ciprofloxacin promotes cell death by converting Topoisomerase II to cellular poison [23]. Norfloxacin induced cardiac hyperplasia. This result agreed with that; animal experiments as well as clinical experience show that the cardiotoxic potentials of sparfloxacin and grepafloxacin are higher than those of the other fluoroquinolones: they cause QT prolongation at rather low doses thus increasing the risk for severe arrhythmia [24]. This lesion might be attributed to ability of fluoroquinolones to block cardiac potassium channel which led to prolonged QT interval with cardiac arrhythmia and consequently cardiac hyperplasia [25]. Pulmonary hypoplasia might be attributed to extensive distribution into lung and achieved higher concentration [26]. Norfloxacin administration induced hypoplasia or atrophy of one or both kidneys. These results agreed with those reported after administration of ofloxacin to rats and rabbits [27].

Oral administration of norfloxacin produced some skeletal malformations such as impairment of skull ossification, absence or dislocation of sternebrae, reduction or absence of caudal vertebrae, and absence of digit's bone of fore- and hindlimb with absence of some metatarsal and metacarpal bone. These skeletal malformations agreed with that recorded by many investigators following administration of ofloxacin to female pregnant rats and rabbits [18]; administration of levofloxacin to rats [19], and administration of fluoroquinolone (DW-116) to the pregnant rats and rabbits, respectively [28, 29]. Fluoroquinolone antibiotics are associated with a wide spectrum of musculoskeletal complications that involve not only tendon but also cartilage, bone, and muscle [30]. Fetal growth retardation could occur as a result of reduction of thickness in proliferative zone of the long bones and absence of the hypertrophic zone. Fluoroquinolone delayed the developmental phase of the epiphyseal growth with growth inhibition [31]. Bone and cartilage damage could be due to fluoride accumulation with repeated fluoroquinolone administration [32]. The fetotoxic effect of ciprofloxacin was observed on skeletal growth as evidenced by decrease of intact bone length in long bones of extremities [17]. The fetotoxicity, high resorption ratio, and fetal loss and malformations could be attributed to the inhibition of DNA transcription in the rapidly divided fetal cells. So fluoroquinolones act as DNA gyrase inhibitors and also mitotic inhibitors. The complete damage of DNA could result in fetal loss or resorption, while partial damage could induce fetal malformation [33].

## 5. Conclusion

Administration of norfloxacin during pregnancy especially in early stage and at high doses could induce some fetal defects and abnormalities, so it is advisable to avoid using this drug during pregnancy.

## Figures and Tables

**Table 1:** Effect of norfloxacin on fetuses obtained from pregnant female rats after repeated oral administration of 500, 1000, and 2000 mg norfloxacin/kg·b·wt from 6th to 15th day of pregnancy once daily (*n* = 10).

Parameters	Control group	500 mg/kg·b·wt (A)	1000 mg/kg·b·wt (B)	2000 mg/kg·b·wt (C)
Number of female rats	10	10	10	10
Number of viable fetuses	91 (100%)	80 (98.88%)	61 (89.71%)	39 (68.42%)
Number of dead fetuses	0	0	2 (2.94%)	7 (12.28%)
Number of resorbed fetuses	0	1 (1.23%)	5 (7.35%)	11 (19.30%)
Total used	91	81	68	57
Fetal body weight (gm)	4.36 ± 0.79	3.71 ± 0.68	3.19 ± 0.54	2.78 ± 0.47
Fetal crown-rump length (cm)	4.29 ± 0.64	3.74 ± 0.71	3.28 ± 0.61	3.01 ± 0.59

%: percent in relation to the total number of examined fetuses.

**Table 2:** Visceral abnormalities in fetuses obtained from pregnant female rats after repeated oral administration of 500, 1000, and 2000 mg norfloxacin per kg·b·wt once daily from 6th to 15th day of pregnancy once daily (*n* = 15).

Parameters	Control group	500 mg/kg·b·wt (A)	1000 mg/kg·b·wt (B)	2000 mg/kg·b·wt (C)
Number of examined fetuses	15	15	15	15
Brain diverticulum	—	8 (53.33%)	9 (60%)	12 (80%)
Thymus hypoplasia	—	6 (40%)	8 (53.33%)	9 (60%)
Lung hypoplasia	—	9 (60%)	9 (60%)	11 (73.33%)
Heart enlargement	—	8 (53.33%)	9 (60%)	12 (80%)
Liver enlargement	—	10 (66.67%)	12 (80%)	14 (93.33%)
Suprarenal gland enlargement	—	7 (46.67%)	8 (53.33%)	11 (73.33%)

%: percent of total abnormalities in relation to the number of examined fetuses.

**Table 3:** Skeletal abnormalities in fetuses obtained from pregnant female rats after repeated oral administration of 500, 1000, and 2000 mg norfloxacin/kg·b·wt from 6th to 15th day of pregnancy once daily (*n* = 15).

Parameters	Control group	500 mg/kg·b·wt (A)	1000 mg/kg·b·wt (B)	2000 mg/kg·b·wt (C)
Number of examined fetuses	15	15	15	15
Impairment of skull ossification	—	3 (20%)	5 (33.33%)	8 (53.33%)
Absence or dislocation of sternebrae	—	2 (13.33%)	3 (20%)	5 (33.33%)
Reduction or absence of caudal vertebrae	—	5 (33.33%)	6 (40%)	10 (66.67%)
Absence of digit's bone of fore limb	—	3 (20%)	4 (26.67%)	9 (60%)
Absence of digit's bone of hind limb	—	2 (12.33%)	3 (20%)	7 (46.67%)
Absence of some metatarsal bone	—	3 (20%)	4 (26.67%)	6 (40%)
Absence of some metacarpal bone	—	2 (12.33%)	4 (26.67%)	5 (33.33%)

%: percent of total abnormalities in relation to the number of examined fetuses.
